# What factors are associated with the research productivity of primary care researchers in Canada? A qualitative study

**DOI:** 10.1186/s12913-024-10644-6

**Published:** 2024-03-01

**Authors:** Monica Aggarwal, Brian Hutchison, Sabrina T. Wong, Alan Katz, Steve Slade, Deirdre Snelgrove

**Affiliations:** 1 University of Toronto, Dalla Lana School of Public Health, Toronto, Ontario Canada; 2 McMaster University, Departments of Family Medicine and Health Research Methods, Evidence and Impact and the Centre for Health Economics and Policy Analysis, Hamilton, Ontario Canada; 3 University of British Columbia, School of Nursing, Centre for Health Services and Policy Research, Vancouver, British Columbia Canada; 4 University of Manitoba, Departments of Community Health Sciences and Family Medicine Rady Faculty of Health Sciences, Winnipeg, Manitoba Canada; 5 The College of Family Physicians of Canada, Mississauga, Canada

**Keywords:** Primary care, Research productivity, Qualitative study, Individual, Institution, System, Professional

## Abstract

**Background:**

Research evidence to inform primary care policy and practice is essential for building high-performing primary care systems. Nevertheless, research output relating to primary care remains low worldwide. This study describes the factors associated with the research productivity of primary care researchers.

**Methods:**

A qualitative, descriptive key informant study approach was used to conduct semi-structured interviews with twenty-three primary care researchers across Canada. Qualitative data were analyzed using reflexive thematic analysis.

**Results:**

Twenty-three primary care researchers participated in the study. An interplay of personal (psychological characteristics, gender, race, parenthood, education, spousal occupation, and support), professional (mentorship before appointment, national collaborations, type of research, career length), institutional (leadership, culture, resources, protected time, mentorship, type), and system (funding, systematic bias, environment, international collaborations, research data infrastructure) factors were perceived to be associated with research productivity. Research institutes and mentors facilitated collaborations, and mentors and type of research enabled funding success. Jurisdictions with fewer primary care researchers had more national collaborations but fewer funding opportunities. The combination of institutional, professional, and system factors were barriers to the research productivity of female and/or racialized researchers.

**Conclusions:**

This study illuminates the intersecting and multifaceted influences on the research productivity of primary care researchers. By exploring individual, professional, institutional, and systemic factors, we underscore the pivotal role of diverse elements in shaping RP. Understanding these intricate influencers is imperative for tailored, evidence-based interventions and policies at the level of academic institutions and funding agencies to optimize resources, promote fair evaluation metrics, and cultivate inclusive environments conducive to diverse research pursuits within the PC discipline in Canada.

**Supplementary Information:**

The online version contains supplementary material available at 10.1186/s12913-024-10644-6.

## Background

In 2008, the World Health Organization highlighted the importance of producing knowledge and research to accelerate primary care (PC) reform [1]. Research evidence to inform PC policy and practice is essential for high-performing PC systems [2]. Despite this recognition, research output in 21 countries found PC research represented a small proportion of total publications as of 2017 [3]. This trend may be due to greater investments in laboratory and specific disease conditions research than PC research focusing on front-line patient and community care [4]. Past work has also shown that clinical investigators have the highest scientific productivity (Canadian Institutes of Health Research (CIHR) funding rates, bibliometric indicators of impact) and health services and population health researchers have the lowest [5]. However, PC researchers, many of whom are clinicians, are also health services researchers and carry out research using participatory action and community based approaches [6] which could subsequently affect bibliometric indicators of impact. 

There is extensive literature examining the factors associated with the research productivity (RP) of researchers across countries [7–13] and disciplines [14–18]. Our literature review on “research productivity” in PubMed yielded 3,456 abstracts that were reviewed for relevancy. This literature illustrates that RP (defined through metrics such as the number of publications, number of citations, and h-index) is influenced by individual, professional, institutional, and system-level factors [19] (Refer to Table 1). 

**Table 1:** Literature on Factors Associated with Research Productivity

Level of Factors	Factors associated with RP
Personal	• Gender [15, 20] • Race [20] • Age [15, 20] • Language [8] • Marital status [15, 20] • Having dependent children [20] • Number of children [20] • Spousal occupation [21] • Education (PhD program and ranking) [15, 20] • Years to complete the degree [20] • Dissertation subfield [20] • Psychological and cognitive characteristics: genuine interest in one’s discipline or field [20, 22], socialization [23], professional commitment [16, 22, 23], motivation [15, 20, 22, 23], and desire for recognition [20].
Professional	• Academic rank [15, 16, 20, 24] • Academic discipline [24] or expertise [23] • Subfield specialization [20] • Tenure status [22], employment school ranking [20] • Frequency of conference presentations [20] • Research experience since completion of PhD [20] • Collaboration with other researchers (co-authorship) [20] • Academic career satisfaction [24].
Institutional	• Private or public institutions [20] • MA or PhD granting institution [20] • FTE student-to-faculty ratio [15, 24], Size/experience/expertise [15, 23] • Prestige or rank of the department [20]. • A working environment with a culture [23] of shared attitudes about the value of research [16, 22] • Collegiality and interpersonal encouragement [20], communication [23] • Resource [23] • Incentive opportunities [23]
System	• Importance of PC in the health care system and universities, training and funding of PC researchers [3] • Strong professional and academic colleges [3] • National data collection network [3] • Organizing PC research teams [3] • Favourable conditions for publishing in English [3] • International research networks [3] • COVID-19 pandemic [25]; [25–28] • Systemic bias of racialized researchers, particularly women of colour [25, 29–32].

Although there is a vast literature on the factors that shape RP, little is known about the factors that influence the RP of PC researchers. Understanding these factors becomes paramount in enabling robust, evidence-driven policies and practices that cater to the diverse and intricate needs of PC researchers. Identifying influential factors aids in directing resources efficiently, allowing institutions and funders to invest in areas that foster higher RP amongst researchers. This allocation may include funding support, infrastructure development, and targeted initiatives, such as peer support [33, 34]. Likewise, by comprehending the determinants of productivity, institutions and funders can develop more holistic and fair evaluation metrics [33]. This ensures assessments consider various measures of RP beyond traditional publication metrics [35] and promote work-life balance, address systemic biases, and nurture inclusive environments conducive to diverse research pursuits [36, 37].

In the PC discipline, two types of researchers are involved in PC research. This includes clinicians who often have limited time to conduct research and PhD-trained researchers who are not clinicians but have teaching loads. In the context of exploring the factors associated with the RP of PC researchers, a qualitative study stands as an indispensable methodology to delve deeply into intricate and multifaceted phenomena by facilitating an in-depth comprehension of individual, professional, institutional, and systemic determinants, shedding light on the intricacies that quantitative analyses might overlook [38, 39]. This is the first study to examine the individual, professional, institutional, and system factors that influence the RP of PC researchers in Canada.

## Methods

### Study design

We employed a qualitative, descriptive study, with no predetermined theoretical framework, using key informants since it is the most effective method to comprehensively describe participant perceptions of a specific phenomenon [40, 41]. We used the Consolidated criteria for Reporting Qualitative Research to report this study [42] (Supplementary Material 1). Ethics approval was obtained from the University of Toronto (#43,254).

### Setting and participants

Key informant interviews were conducted with Canadian PC researchers. Potential participants were emailed. The majority of those emailed participated. We conducted purposeful sampling using a demographic survey to identify and contact PC researchers with diverse backgrounds based on gender, ethnicity, parenthood, research expertise, education, credentials (e.g., MD versus PhD), profession (physicians, nurses), academic rank, jurisdiction, and career length [43]. Theoretical sampling was employed to understand the variation between researchers’ experiences of productivity related to concepts such as their sex/gender, racialization, profession, and researcher type (clinician or PhD-trained). The researchers created a participant list by reviewing a pre-existing list of PC researchers across 13 jurisdictions in Canada. Participants with experience related to these categories were recruited via email as the analysis occurred. Five participants declined interviews due to constraints on their time. Purposive sampling considered the concept of theoretical saturation, meaning we included new participants until the interviews did not yield new information to meet the original objectives [41, 44, 45]. The Principal Investigator (MA), a female PhD-trained qualitative researcher, recruited participants through an email invitation with study details. Three reminders were sent to potential participants. All participants provided written consent and verbal permission. MA did not have pre-existing personal relationships with interviewees. The participants were informed about the reasons for conducting the research.

### Data collection

Sixty-minute semi-structured telephone or virtual video interviews were conducted to explore PC researchers’ perspectives on their RP [38]. No pilot interviews occurred. These interviews were audiotaped, transcribed verbatim, and reviewed for accuracy. Before the interview, participants completed a demographic questionnaire. During interviews, respondents were asked broad questions about the individual, professional, institutional, and system factors that shaped RP, barriers and facilitators, and lessons learned (refer to Supplementary Material 2 for Interview Guide). In line with Marshall and Rossman’s recommendations, the interview guide purposively asked open-ended questions to provide ample opportunities for participants to convey knowledge [46]. The PI took field notes during the interview.

### Data analysis

Reflexive thematic analysis was performed simultaneously with data collection to determine data saturation [47]. Demographic questionnaire data were analyzed using descriptive statistics. Qualitative data were analyzed using questionnaire data and thematic analysis proposed by Braun and Clarke [48, 49]. First, transcripts were read and re-read for data familiarization by two research assistants (ZH and CW) and the PI. The first five transcripts were independently coded separately by two RAs. Codes were arranged into themes and sub-themes using thematic maps to organize related concepts. Codebook thematic analysis was chosen as it retains the flexibility offered by ‘reflexive’ thematic analysis, allowing analysis to begin deductively (using the topic guide and themes from existing literature) but become increasingly inductive as deeper engagement with the data offers novel insights [50–52]. After analysis of five transcripts, the RAs and PI met to discuss emerging themes and subthemes and develop a coding framework (codebook). The coding framework was tested on two transcripts to develop a final codebook. This coding framework was then used for all the transcripts including re-analysis of the first five transcripts. The codebook included inductive codes that emerged through the data such as individual attributes (e.g., skills, motivations), professional influences (e.g., collaboration, training), institutional factors (e.g., resources, support), and systemic elements (e.g., policies, external environment).

To increase the transparency and rigour of the process, two RAs used the coding framework to independently code the data line-by-line on the remaining transcripts. We used an iterative process to move codes in and out of categories until a hierarchy of codes, subthemes, and themes was established. The data coded by RAs were reviewed for consistency, and discrepancies were addressed through discussion and data review to inform a collective decision. The research team wrote reflexive notes throughout data collection and analysis to increase transparency [53, 54]. The coding process was facilitated using NVivo 12.0 [55].

After the data were coded, RAs reviewed the data and developed summaries for each theme and sub-theme as suggested by the DEPICT method [56]. Discussion about the data was ongoing between the team to ensure the qualitative analysis was trustworthy [57] and to develop a shared set of themes. Alternative interpretations of the data were discussed. Each RA re-reviewed the data to confirm the themes matched our understanding of the data, with attention to code occurrences. This phase resulted in a rich descriptive analysis and write-up of the participants’ perspectives. After analyzing and finalizing the themes and sub-themes, a visual representation of the findings was developed. Member checking was conducted with two researchers who agreed with the findings.

### Rigour

Our methods aimed to adhere to the principals of procedural and analytical rigour [58]. Employing purposeful and theoretical sampling techniques facilitated a diverse participant pool, enhancing the dependability and transferability of our findings by encompassing varied backgrounds and experiences relevant to research productivity. Our analysis approach, involving multiple coders and iterative discussions among the research team, fostered triangulation and enhancing the trustworthiness of our qualitative analysis. The integration of member checking further fortified the credibility and dependability of our findings.

## Results

### Participant characteristics

Twenty-three PC researchers participated in the study. Table 2 provides information on participant characteristics.

**Table 2:** Characteristics of Participants

Demographic Characteristics	Count	%
Gender		
Female	13	57%
Male	10	43%
Racialization		
Not Racialized	14	61%
Racialized	8	35%
No response	1	4%
Education/Credentials		
PhD	8	35%
PhD & MD	8	35%
MD	5	22%
Other PhD & MD (Combined and PhD & NP/RN	2	8%
	1	4%
Marital Status		
Married	18	78%
Other	5	22%
Dependent Y/N		
Yes	16	74%
No & No response	7	26%
Age Group		
<35 years old	2	9%
35–49 years old	8	35%
50–64 years old	4	17%
65 < years old	6	26%
No response	3	13%
Profession		
Physician	13	56%
Nurse (RN, NP)	3	13%
Not a healthcare provider	6	26%
No response	1	4%
Years as Independent Researcher		
<11 years	3	13%
11–15 years	4	17%
>15 years	10	43%
No response	6	26%
University Affiliation		
University of British Columbia	1	4%
University of Alberta	1	4%
University of Calgary	2	9%
University of Saskatchewan	2	9%
University of Manitoba	1	4%
University of Toronto	4	17%
Western University	3	13%
McMaster University	1	4%
University of Ottawa	2	9%
Université Laval	1	4%
University of Sherbrooke	1	4%
McGill University	2	9%
Dalhousie University	1	4%
Memorial University	1	4%
Department Affiliation		
Family Medicine	21	75%
Nursing / Public Health	2	8%
Jurisdiction		
British Columbia	1	4%
Alberta	3	13%
Saskatchewan	2	9%
Manitoba	1	4%
Ontario	12	52%
Quebec	4	17%
Nova Scotia	1	4%
Newfoundland and Labrador	1	4%

### Factors that impact research productivity

**Fig. 1:**
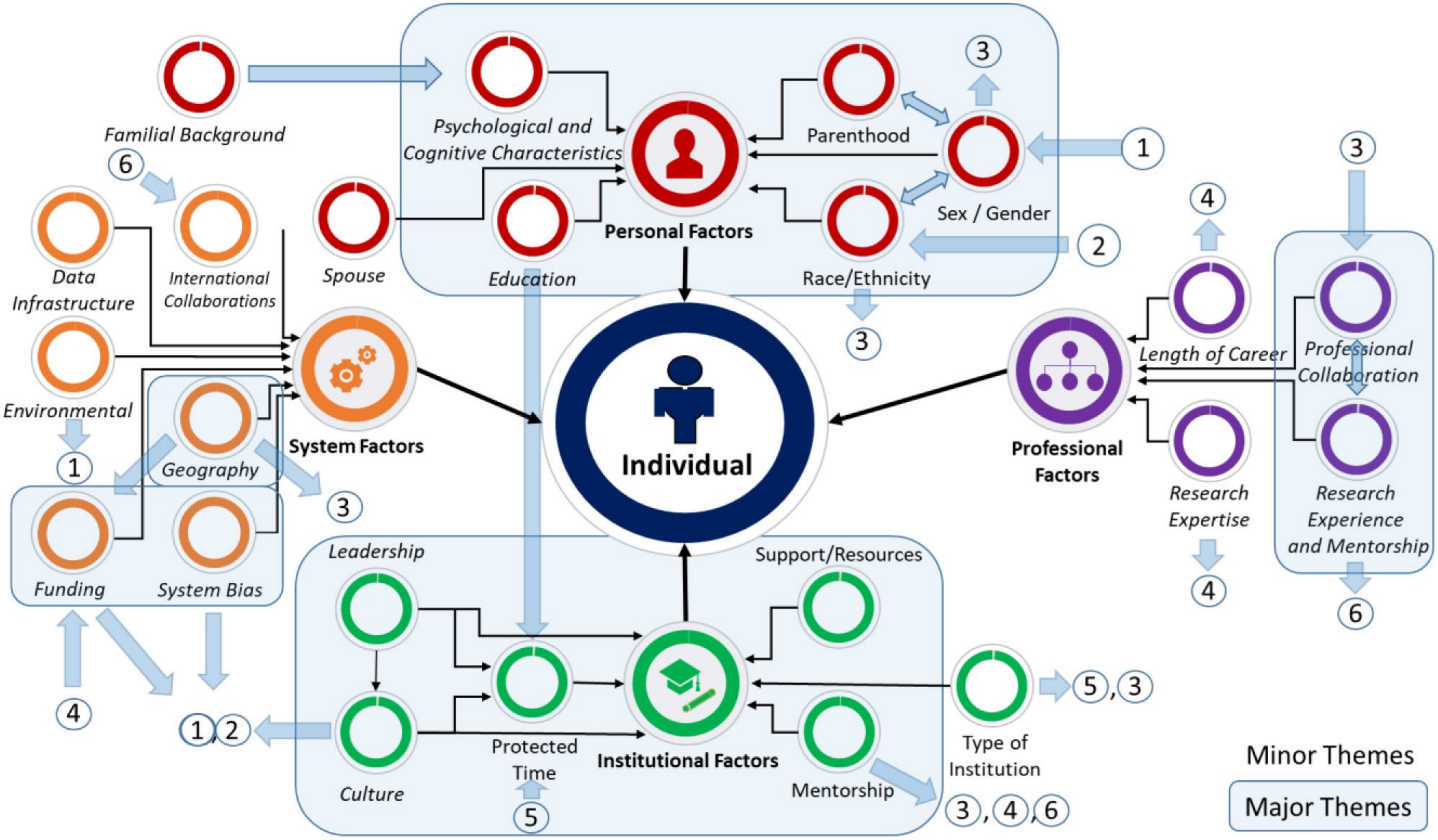
Factors that determine research productivity in primary care. 1: Factor impacts sex/gender. 2: Factor impacts sex/gender and race/ethnicity. 3: Factor impacts professional collaboration. 4: Factor impacts funding. 5: Factor impacts protected time. 6: Factors impacts international collaboration

Key informants identified various individual, professional, institutional and system factors that impacted their RP and discussed how these factors impeded or served as facilitators to their RP. Fig. 1 - **Factors that Determine Research Productivity in Primary Care** demonstrates the interplay between these factors and the important role of institutions and funders in facilitating or hindering RP. Supplementary Material 3 provides additional illustrative quotes for the themes.

### Personal factors that impact research productivity

Personal factors that emerged as major themes included gender, parental status, race/ethnicity, educational background, and cognitive and psychological characteristics. Sub-themes included spousal occupation and support. Psychological characteristics, spousal occupation and support were perceived to facilitate RP, whereas having children, being a female, being a racialized researcher, or being a physician (MD) researcher was a barrier. Some researchers noted that their familial background influenced their psychological and cognitive characteristics.

### Professional factors that impact research productivity

Collaborative networks, research expertise, and mentorship were major themes and length of career was a sub-theme. Collaborative networks between researchers facilitated RP and benefited clinical researchers without graduate degrees. Alternatively, collaborations were barriers when team members were unreliable or did not contribute or when they excluded racialized researchers (number 3, Fig. 1).

The type of research conducted by PC researchers was identified as a facilitator when it aligned with policymakers’ priorities, as it enabled funding opportunities and RP. On the other hand, it was a barrier for *“methodological researchers”* whose research did not benefit policymakers (number 4, Fig. 1).

Mentorship before faculty appointment was crucial to RP and was described as being related to professional (collaboration) and system (funding) factors (numbers 3, 4 and 6, Fig. 1). A researcher said: *“Great mentors who are in the position to be able to open doors for you, I think definitely affected my productivity, because then it supports me to get grants, they kind of hand me data that gives me publications through their project.”* (Researcher 7).

Length of career was a sub-theme that facilitated and served as a barrier to RP, depending on the career stage. Established researchers were more likely to succeed in grant competitions than early-career researchers, increasing RP (number 4, Fig. 1).

### Institutional factors that impact research productivity

Institutional resources and support for research, protected time for research, mentorship, leadership, and research-supportive culture surfaced as major themes when discussing factors influencing productivity. These factors were perceived as barriers or facilitators of RP, depending on their presence or absence.

Institutional resources and supports (e.g., project funding, research chair positions and administrative support) were key facilitators for RP. A participant noted it is not enough for leaders to *“hire somebody and then put them in an empty office. I think most organizations would understand that you need to build an infrastructure to help. So, if you were hiring a senior faculty member, or even a junior faculty member, and then that person is put in place without a support structure, administrative supports, somebody to help with research assistance, those kinds of things, I don’t think it’s going to work. That’s just setting people up to fail.”* (Researcher 14).

Leadership was also identified as a barrier or facilitator to RP, depending on whether departmental leaders were committed to PC research. A participant: “*We suddenly had a dean of medicine who didn’t want to pay attention to [research] and wanted 60% to be the maximum amount that researchers were allowed to spend on research.*” (Researcher 19). Protected time for research was identified as a major factor for determining RP and often identified as a barrier for MD-trained researchers.

Researchers also noted that institutional culture, which is driven by the leadership, can be a facilitator or barrier to equity (women, parenthood, minorities), research discipline (clinical versus health services), and support for clinical and non-clinical faculty. Female researchers described the prevailing culture in their departments as driven by a *“boy’s club”* in which women are expected to do more to succeed or be considered for leadership positions (number 1,2, Fig. 1). Racialized female researchers described a culture of “exclusion” and feeling their “voice” was not heard.

Mentorship was a major theme, with many participants crediting their career success to their mentors (3,4, Fig. 1). *“So, I think for me, probably the single most impactful attribute that has led to my productivity has been being super lucky to have extremely great mentors. [My mentor] continued and continues to be one of my most meaningful and impactful mentors, and we’ve done tonnes of work together. And even some work that I’ve done that [my mentor] [has] not been on, [he has] been involved with in terms of...coaching and putting me in contact with some people, or...helping sort of orient or support. I would say mentorship has been tremendous.”* (Researcher 3).

Employment at a research institute (i.e., a not-for-profit corporation that employs scientists) was a sub-theme that facilitated collaborations, access to data and protected time for research, increasing RP (numbers 5 and 3, Fig. 1).

### System factors that impact research productivity

System factors that served as major themes included funding, systematic bias, and geography (jurisdictional location of a researcher). Sub-themes included environment (contextual factors (COVID-19 pandemic)), international collaborations, and research data infrastructure. Researchers indicated that investments in PC research were important for obtaining grants for RP. Some participants indicated that because PC is not disease-focused, the lack of funding opportunities for researchers is a significant barrier:

*“For primary care [or] family physicians, there’s no industry for us compared to some specialty areas or some other people in other areas. For example, I see [a researcher that] has lots of grants, but all of them are matched with industry. So that’s where you bring money from industry...usually for National Research Council Canada, they match the money. Then you come up with a $2 million grant. But I mean for us... I mean in medicine, it’s less. But still, in primary care, it is almost zero.”* (Researcher 2).

Geography positively or negatively impacted funding opportunities and professional collaborations (number 3 in Fig. 1). Geographies with fewer PC researchers enabled collaboration but resulted in less access to funding opportunities. A participant noted:

*“So we don’t have a provincial funding agency. So there are small pockets, very small pockets, relatively speaking, available through the university….The largest grants I think we have through the university are $25 or $30,000. So that can help to establish a bit of a track record in research. There’s a large jump between that and a typical Canadian Institutes for Health Research (CIHR) grant… So making the step-wise or stepping up to competing at CIHR is the challenge.”* (Researcher 9).

Participants from a French-speaking jurisdiction indicated being francophone impacts research since linguistic barriers require resources for translation for English-speaking journals, conferences, and grant applications and can serve as a barrier to collaborations. On the other hand, being an anglophone in a francophone environment facilitated RP and success since it enabled inclusion on research teams and across the country.

Several researchers indicated systemic racism in research as a barrier to RP. Participants talked about how unconscious biases are woven into institutional cultures at different levels, which results in *racism* because of sexism, which directly impacts female and/or racialized researchers at the institutional and system level (numbers 1, 2, Fig. 1). A non-racialized researcher indicated: “*When I listen to faculty members… from different racial groups….I think that there are more challenges for them… I think some of it is longstanding racism that they’ve experienced that’s had an influence [on RP].”* (Researcher 23). A racialized female researcher described her experiences with leadership and colleagues: *“People in leadership who were great mentors would literally walk by me in the hall-way and just really had not acknowledged me...I heard of others having quite different experiences. I do think that for me, being a woman and one of colour played a role in that.”* (Researcher 7).

The COVID-19 pandemic was a significant environmental factor that reduced RP due to the challenges for female researchers with children who could not *“apply quickly to grants because [they] had to manage school duties, kindergarten”* (Researcher 11) and there were challenges posed for colleagues directly involved in patient care (number 1, Fig. 1).

International collaborations contributed to RP. It allowed one to learn and understand researchable problems and different healthcare systems across a wide range of socioeconomic realities and different cultures.

Lastly, it was noted that access to a research data infrastructure (creation of national datasets that collect information for research) positively impacted RP. In contrast, others indicated:

*“There is a need to reduce barriers to data access, especially across provincial boundaries. Down in the States, they can do so much with national datasets that we’re just so hamstrung here. And it’s really ridiculous, the barriers.”* (Researcher 9).

## Discussion

PC research is often conducted in higher education institutions and is fundamental for informing evidence-based decision-making and building high-performing PC systems [59]. Notably, despite the acknowledged significance of primary care reform by the WHO [1], the advancement of knowledge in this domain remains a crucial yet relatively underrepresented facet within the research landscape. While PC serves as the cornerstone of healthcare systems, the paucity of research investment and output directed towards front-line patient and community care is limited [3, 4]. This discrepancy in research output is compounded by a lack of exploration into the specific factors influencing the RP of PC researchers in Canada. Similar to the international literature, we found that a variety of key personal [8, 15, 16, 20–23], professional [15, 16, 20, 22–24], institutional [15, 16, 20, 22–24], and system factors [3] impact the RP of PC researchers. While international literature indicates RP is similar between research institutes and universities or higher in universities [60, 61], we found, in contrast, that research institutes enhance RP. This might be explained by more funding for research [60, 61] and protected time for researchers at institutes, compared to those at universities that might have teaching and administrative requirements.

This study’s major contribution is showing how the interplay of different factors impacts RP. For example, working at a research institute positively impacted collaborations and protected time. In turn, collaborations and protected time were perceived to increase RP. Mentors were perceived to foster national and international collaborations and success in funding competitions, consequently increasing RP. Research expertise benefiting policymaking enabled success in funding opportunities, impacting RP. Jurisdictions with fewer PC researchers were more involved in national collaborations but had fewer funding opportunities, positively or negatively impacting RP. Finally, this study shows how the combination of institutional (inequitable culture), professional (exclusion by colleagues), and system (systemic bias, pandemic situations, lack of success in funding) factors negatively impact the productivity of female and/or racialized researchers.

Funders and academic institutions are critical in supporting and accelerating RP. At the institutional level, departments of family medicine, schools of nursing and community health sciences should recruit effective leaders committed to PC research who will cultivate supportive, flexible, and equitable cultures for researchers [62, 63]. These leaders should establish a clear statement of vision and mission statements, and strategic plans, thus sending a clear signal of commitment to researchers and RP [64]. This should be accompanied by investments in research and administration (i.e., financial, infrastructure and human capital) [64, 65]. Funding can be provided for conferences, project support, publication fee support, incentives for publication, recognition of research achievements, fellowships, workshops and training, and writing retreats [66]. Infrastructure support may include more research centers, laboratories, co-working spaces [64] and librarian services [67]. Human capital support includes promoting self-efficacy, research skills and competence, and global innovativeness through support for training and workshops [64]. Furthermore, allowing dedicated protected time is critical for RP [64].

To address the complex needs of faculty members who are academics interested in PC or clinician scientists delivering PC, there is a need for formalized mentorship programs [68, 69] that emphasize the importance of reciprocal learning, emotional support, career guidance, and work-life balance in mentoring relationships [70, 71]. The reciprocal nature of mentoring relationships between trainees and early career faculty and senior faculty can create a mutually beneficial outcome of RP for both mentors and mentees. Thus, institutions can assist by strategically pairing senior faculty with junior faculty and students in the same field of study [66]. Mentorship is particularly important for racialized researchers, who we and others have found are more likely to face barriers to resources, exclusionary practices, and stereotypes [32, 72].

At the individual level, trainees and early career faculty are important in facilitating their own RP. Individuals can choose supportive working environments, seek out different mentors and colleagues that respectfully support their career goals [73], reach out to obtain institutional support and engage in professional networks (e.g. primary care research network) that will permit the meeting of colleagues, facilitating collaborations and accessing data for research.

At the systems level, governments and granting agencies should consider targeted funding for PC research and training and support a national data infrastructure to enable the continuous flow of PC research. Strategies must be implemented at all levels to address gender and racial inequalities, which will be addressed in a separate paper.

### Limitations

A limitation of this study is that it does not explicitly explore the diverse research approaches (e.g., engaged scholarship, participatory action research) and their distinct time and relationship demands, potentially limiting the comprehensive exploration of nuanced research domains [5]. Importantly, RP is an indicator of academic performance which may not be as important to communities as shortening the time of spreading innovations among PC practices trying to improve the quality of care. In addition, the use of purposeful sampling, while aiming for diversity across various demographics and professional backgrounds, may have inadvertently excluded perspectives not captured within the selected sample. This could limit the transferability of findings. While member checking was performed with a subset of researchers, the extent to which their perspectives represent the entire participant cohort might not have been fully understood, potentially affecting the robustness of the identified themes [74].

## Conclusion

This study illuminates the multifaceted determinants influencing the RP of PC researchers and demonstrates that an interplay of individual, professional, institutional, and systemic factors influence the RP of PC researchers.

Our study underscores the pivotal role of funders and academic institutions in supporting and accelerating RP among PC researchers. At the institutional level, recruiting leaders committed to PC research and fostering supportive, flexible, and equitable cultures is vital. Investments in research infrastructure, dedication to protected time, and formalized mentorship programs emerged as critical pillars. Governments and granting agencies should consider targeted funding for PC research and strategies, investing in training, bolstering a robust national data infrastructure and implementing strategies to improve equity. Trainees and early career faculty would benefit from choosing supportive environments and seeking diverse mentors and colleagues aligned with their career goals.

A forthcoming paper will delve deeper into these critical issues, proposing interventions essential for fostering a more inclusive and equitable landscape for PC researchers. This includes examining the impact of systemic biases and delineating actionable strategies at institutional and systemic levels to bridge existing gaps and nurturing a more diverse and thriving PC research community.

### Electronic supplementary material

Below is the link to the electronic supplementary material.

Supplementary Material 1

Supplementary Material 2

Supplementary Material 3

## Data Availability

The manuscript includes the data and study material.

## Declarations

### Authors’ information

The findings of this article are those of the authors. They do not necessarily reflect the views of their affiliated institutions, employers.

## Abbreviations

CIHR: Canadian Institutes of Health Research
PI: Principal Investigator
PC: Primary Care
RA: Research Assistant
RP: Research Productivity
