# Supplementary material for: What factors are associated with the research productivity of primary care researchers in Canada? A qualitative study
Source: BMC Health Serv Res. 2024 Mar 1;24:263. doi: 10.1186/s12913-024-10644-6 (PMC10908166; doi:10.1186/s12913-024-10644-6)
Supplement: Supplementary file 2 — Supplementary Material 2 [file 12913_2024_10644_MOESM2_ESM.docx]

**Supplementary Material 2: Interview Guide**

1. Please describe your contribution to primary health care research. How has your research evolved over time?
2. How would you define “productive”, in terms of research productivity?

*Probe:* Considering your work experiences, what is expected of you in terms of “productivity” ? Should measures/definitions of productivity consider the role of sex/gender or race? If so why/why not. If so, how?. How could racialized or diverse-gender individuals be better supported by their organizations to reach measures of productivity?

1. In your view, what factors had the most significant impact – either positive or negative - on your research productivity?

*Probe for:*

- *Individual Factors:* Demographics*,* education, years to complete the degree, and dissertation subfield, psychological and cognitive characteristics
- *Professional Factors:* Academic faculty rank, academic discipline, subfield specialization, tenure status, employment school ranking, frequency of conference presentations, research experience since completion of PhD, collaboration with other researchers and academic career satisfaction.
- *Institutional Factors:* Master’s or PhD granting institution, FTE student-to-faculty ratio, size/experience/expertise, and quality, prestige or rank of department, culture, resources, mentorships, staff support, well-developed network of colleagues outside the department with whom to discuss research and education, time to do research, less time on teaching and courses, committee service, advising students, enough faculty in department to achieve goals, tenure and promotion, satisfying performance standards, and peer and social recognition, leadership
- *System Factors:* the importance of PC in the health care system and universities; training and funding of PC researchers; strong professional and academic colleges; structuring of a national data collection network; structuring of PC research teams; favorable conditions for publishing in English; and international research networks and cooperation

1. Do you think there are gender differences in experiences related to research productivity? If so, could you please describe or share a story?
2. Do you think there are racial differences in experiences related to research productivity? If so, could you please describe or share a story?
3. Do you think there are differences in experiences based on credentials (MD versus PhD researcher) related to research productivity? If so, could you please describe or share a story?

*Probe: If you’re a clinician researcher, how do you protect time for research activities? What challenges do you face in trying to protect time for research? What helps you protect time from research?*

*Probe for MDs: How do you balance clinical work (e.g.., responsibility for patient panel) and research?*

*Probe for educators: How do you balance teaching and research?*

1. Based on your experience and observations, what factors serve as facilitators of research productivity?
2. What factors served as barriers to research productivity?
3. What advice would you give to the College of Family Physicians of Canada on how to support grown of primary care research?
4. What advice would you give to the institutions (e.g., universities, medical schools, faculties of health science) on how to foster research productivity of their primary health care researchers?
5. What advice would you give to departments of family medicine, schools of nursing and other health related university departments on how to inspire and nurture the development of primary health care researchers?
6. What advice would you give to colleagues on how to help foster research productivity of students and trainees?
7. If you were mentoring an aspiring or early career primary health care researcher, what advice would you give them about preparing for a career in academia?
