# Supplementary material for: What factors are associated with the research productivity of primary care researchers in Canada? A qualitative study
Source: BMC Health Serv Res. 2024 Mar 1;24:263. doi: 10.1186/s12913-024-10644-6 (PMC10908166; doi:10.1186/s12913-024-10644-6)
Supplement: Supplementary file 3 — Supplementary Material 3 [file 12913_2024_10644_MOESM3_ESM.docx]

## Supplementary Material 3: Key Informant Quotes on major and minor themes concerning personal, professional, institutional, and system factors

| **Factor** | **Themes/Sub-themes** | **Quotes** |
| --- | --- | --- |
|  | Cognitive and psychological characteristics | Highly productive researchers are “*naturally curious and want to know the answer to things”* (Researcher 1) and they have “*intellectual curiosity.”* (Researcher 10)  Highly productive researchers “*desire to maximally improve the lives of folks in Canada*.” (Researcher 15)  Productive researchers are *“receptive to change and taking feedback from others.”* (Researcher 22).  Productive researchers are “resilient” and “*can deal with rejection*.” (Researcher 13)  *“It doesn't matter if your grant is not funded or your paper gets rejected by this journal or whatever, you’ve just got to like, you know, get up off the ground and just keep doing it, right.”* (Researcher 22) |
|  | Education | Acquiring a PhD degree made a *“massive difference, because [researcher] had as much skill as anyone around the table.”* (Researcher 15).  *“I don't know how much the fact that I don't have a master's or a PhD has impaired my ability to do good research because I've collaborated with good people and have surrounded myself, luckily, with many colleagues who do have some of those advanced degrees.”* (Researcher 3) |
| Professional | Professional collaborations | *“I had another experience where I was asked to start projects with the research and other colleagues. I did quite a bit of work, and I couldn't get any reply. They just wouldn't reply. They wouldn't do any work. They didn't even acknowledge when I sent it….And two other people just didn't even reply……So, it was very disconcerting.”* (Researcher 6) |
|  | Research expertise | *“I want to link just with patient-oriented research….Because if I compare my time and my schedule with my fundamentalist colleague, they have a lot of time to write. Time that I don't have because I have a team meeting with patient partner, with clinician, and I'm collecting data in clinical setting. And so it's a different between fundamental science and primary care science. Yeah, we have to put in a lot of time.”* (Researcher 11) |
|  | Length of career | *“Those individuals who have a long record of publications and very good granting histories…Those who have more, more will be given. Awards are determined by whether the project will be a success.”* (Researcher 14) |
|  | Protected time for research | *“I'd say the biggest institutional support has been time. So having a department that was willing to allocate some time to me, paid, that I could pursue some of these things, of course, has led to being productive, right.”* (Researcher 3)  *“I would say the biggest barriers are my clinical workload. So this 1,200 patient practice that is, you know, just more and more complex over time.”* (Researcher 20) |
|  | Institutional leadership | *“If you want to have a big career in research, your Chair needs to support more time for research.”* Some researchers indicated department leadership was instrumental in supporting a culture of research: *[The] previous chair was in that position for 15 years and really set the tone in a number of ways. Research was very important…, and he was very supportive of it, and also, I think, just how a good perspective on I'm gonna encourage you to do this and make available the resources that you need, and then kind of get out of your way. Like [Chair] wasn’t a micro manager in that way. And he was a very good financial steward of the department which created all of these resources that I think are quite rare.”* (Researcher 16) |
|  | Institutional culture | *“I think the culture of research in most departments of family medicine could be improved….Research, teaching and clinical practice are the three equals. And they must be equal.”* (Researcher 19) |
|  | Type of institution or department/school | “So in the early parts of my career, I was working within an institution, and I was lucky to be working within a pure research institution rather than a single university department, because that meant that I had contact with a small organization with researchers across a wide range of fields, and close collaboration was expected. In a university, I might not have had that.  The institutional correlate of my research experience has been that most of my life as a researcher” (Researcher 14) |
|  | Geography | *“I was in Francophone environments as an Anglophone. And so that was a real help to me because people looked to me to produce. They just thought that that's what I would be doing. And I helped them produce. As I was available to help them with publications, it actually helped me. And I think that being an Anglophone in Quebec, I had connections to the rest of Canada that my Francophone colleagues didn't have. So that put me in touch with kind of like a larger collaborative group. And that was really, really helpful.”* (Researcher 13) |
|  | Research data infrastructure | “*And that's where CPCSSN is great. Because, you know, different jurisdictions have different networks. And so sometimes, a great idea in one network becomes cross-jurisdictional. And so that network of people that are connected and work with each other, you know, suddenly can turn a little small couple of clinic studies into a multi-jurisdictional large study.* (Researcher 3) |
